# Perceptions of Medical Postgraduates in India Toward Pursuing Super Specialty Education: A Cross-Sectional Study

**DOI:** 10.7759/cureus.100768

**Published:** 2026-01-04

**Authors:** Zia Arshad, Ravi Prakash, Anita Rani, Rakesh K Dewan, Rakesh Dixit, Amita Pandey

**Affiliations:** 1 Anesthesiology and Critical Care, King George's Medical University, Lucknow, IND; 2 Anaesthesiology and Critical Care, King George's Medical University, Lucknow, IND; 3 Anatomy, King George's Medical University, Lucknow, IND; 4 Pharmacology and Therapeutics, King George's Medical University, Lucknow, IND; 5 Obstetrics and Gynaecology, King George's Medical University, Lucknow, IND

**Keywords:** career counselling, demographic, lifestyle, medical education, questionnaire

## Abstract

Background

Super specialty (SS) courses such as Doctorate of Medicine (DM) and Master of Chirurgiae (MCh) represent the highest level of medical training in India. Understanding postgraduate (PG) perceptions toward SS is vital for workforce planning and educational reforms.

Objective

To assess the relationship between demographic factors and PG students’ choice of SS, as well as influencing factors, perceived benefits, and barriers.

Methods

A cross-sectional, questionnaire-based survey was conducted among PG residents in medicine, surgery, and anesthesiology at a state medical university. Data on demographics, financial and social factors, and career motivations were analyzed using descriptive and inferential statistics.

Results

Among 63 respondents, 47 (74.60%) were willing to pursue SS. Recognition 20 (31.74%) and academic interest 10 (15.87%) were key motivators. Major barriers included financial constraints 4 (6.34%), either seeking early returns or having loans 32 (50.79%) and bond obligations 46 (73.01%), as well as long training durations 6 (9.52%) among the 17 respondents who were not willing to pursue SS. Critical Care was the most preferred SS 26 (41.27%), especially among anesthesiology residents. No significant associations were found with gender, income, or family medical background.

Conclusion

Despite strong interest, financial burden, training length, and service bonds deter many PGs. Institutional reforms, structured mentorship, and early career guidance are crucial for promoting equitable access to SS training in India.

## Introduction

In India, postgraduate (PG) medical education serves as the cornerstone of specialist training, providing physicians with advanced clinical expertise and professional competence. Beyond this stage, super specialty (SS) programs-such as the Doctor of Medicine (DM) and Master of Chirurgiae (MCh)-represent the pinnacle of medical education and professional development. These programs cultivate highly skilled experts capable of conducting research, driving innovation, and leading within tertiary care settings, thereby addressing the country’s growing need for specialized healthcare services.

There was a noticeable change in students' perceptions at the end of graduation compared to when they entered [1]. The decision to pursue SS training is multifactorial, shaped by personal aspirations, recognition, financial stability, and lifestyle preferences. While many PGs view SS qualifications as a path to professional advancement and prestige, systemic challenges such as limited training opportunities, bond obligations, financial constraints, and prolonged study durations often influence their choices. These factors may deter even highly motivated candidates who aspire to advance their careers in subspecialties.

Previous studies have reported a strong interest in SS training among Indian PGs, with over 80% of respondents expressing intent to pursue further specialization. Preference for branches among graduates is influenced by age-group, sex, and the type of medical school they graduate from [2].

Motivators such as self-esteem, academic growth, and lifestyle enhancement were prominent. Conversely, inadequate mentorship, high financial burden, and lack of institutional support emerged as significant deterrents. Despite policy measures such as reduced qualifying thresholds for entrance examinations, several SS seats remain unfilled annually, reflecting ongoing structural limitations in access and distribution of training.

Understanding PG perceptions toward SS training is crucial for evidence-based workforce planning and medical education reforms in India. The findings may inform targeted interventions to make SS education more equitable, sustainable, and aligned with future healthcare needs.

Study objective

This study, therefore, aims to assess the relationship between demographic variables and the decision to pursue SS courses, to identify influencing factors and perceived benefits, and to analyze barriers that hinder pursuing SS.

## Materials and methods

Study design

A cross-sectional survey was the suitable design as it captures information at a specific point in time and provides a snapshot of the perceptions of medical postgraduates at the time of the study. The target population was medical postgraduates enrolled in King George's Medical University (KGMU). The sampling method chosen was stratified random sampling. This method ensures representation from different specialties and years of postgraduate study.

Data collection was done by a self-administered survey. We developed a questionnaire (pilot testing was conducted with a small group of residents) that captures information on demographics (age, gender, specialty), factors influencing the decision to pursue a super specialty (interest, job prospects, income potential, work-life balance, workload), perceived benefits and challenges of pursuing a super specialty, awareness and access to information about super-specialty courses, preferred mode of preparation for super specialty entrance exams (Appendices 1, 2).

An anonymous feedback form was prepared following discussions with faculty members from medicine, surgery, anaesthesia, and other relevant departments. The feedback form was designed to be a comprehensive document that aims to gather as much information as realistically possible about all factors, both direct and indirect, that can potentially impact the choice of specialty for a medical undergraduate. The students were asked to complete an anonymous feedback form regarding their family and educational background, as well as their prospective fields of specialization and career placement. After that, we administer the questionnaire to a small group of postgraduates to ensure clarity and comprehensiveness. We used online survey tools (e.g., Google Forms) as a data collection platform. The participants in this study are postgraduate medical residents doing Doctor of Medicine (MD)/ Master of Surgery (MS) in KGMU in different specialities. They were sent a questionnaire via Google Forms through email and WhatsApp. They were asked to respond in one week. An extension of a week was given with a reminder to those who didn't respond on time. If they didn't reply in two weeks, then they were excluded from the study.

Data analysis

Data analysis was done using SPSS version 28 (IBM Corp., Armonk, New York). Quantitative analysis used descriptive statistics (frequencies, percentages) to summarize the demographics and responses to various survey questions. Qualitative analysis used open-ended questions are included and analyzed the textual responses thematically to identify emerging patterns and insights. Chi-square test and mean were used. A Likert-type scale was also used for analyzing and interpreting data [3].

Ethical considerations

Obtained informed consent from all participants (Institutional ethics committee King George Medical University issued approval "136 ECM IIA/P20” ). Ensured anonymity and confidentiality of the data collected. Obtained ethical approval from the relevant institutional review board before commencing the study.

By following these methodological steps, we conducted a comprehensive study that provides valuable insights into the perceptions of medical postgraduates regarding the pursuit of super-specialty courses. This cross-sectional, observational study was conducted at a medical university in the state capital. The subjects will be postgraduate residents of anaesthesiology, medicine, and surgery in their third year of the course, to analyze their choice of specialty and the factors associated with it.

## Results

The demographic distribution of the participants (N = 63) included age at joining Bachelor of Medicine and Bachelor of Surgery (MBBS) and PG, gender, marital status, current branch, MBBS percentage, number of National Eligibility cum Entrance Test (NEET) attempts, parental qualification and profession, family income, head of the family, and whether there were doctors in the family. The majority joined MBBS between the ages of 18-20, 12 (19.05%), joined PG at 25-30 years, 23 (36.51%), were pursuing PG, 37 (58.73%), and were almost equally distributed between males, 32 (50.79%), and females, 31 (49.21%). Most were unmarried, 46 (73.02%). MBBS scores between 55-60, 26 (41.27%), and attempted NEET once, 28 (44.44%). The largest parental education group was graduates, 24 (38.10%), most parents worked in jobs or business, 16 (25.40%), 18family income was predominantly <10 lakh, 37 (58.73%), the father was most often head of family, 43 (68.25%), and most had no doctors in the family, 51 (80.95%) (Table 1).

**Table 1:** Demographic and Educational Profile of Study Participants. MBBS: Bachelor of Medicine and Bachelor of Surgery; PG: postgraduate; NEET: National Eligibility cum Entrance Test.

Variable	Categories	Frequency (n)	Percentage (%)
Age at joining MBBS	<18 years	5	7.94
	18–20 years	12	19.05
	>20 years	9	14.29
Age at joining PG	<25 years	4	6.35
	25–30 years	23	36.51
	>30 years	10	15.87
Course	MBBS	26	41.27
	PG	37	58.73
Gender	Male	32	50.79
	Female	31	49.21
Marital Status	Married	17	26.98
	Unmarried	46	73.02
Current PG Branch	Medicine	16	25.39
	Surgery	10	15.87
	Anaesthesia	37	58.73
MBBS Percentage	<55%	7	11.11
	55–60%	26	41.27
	60–65%	18	28.57
	65–70%	7	11.11
	>70%	5	7.94
NEET Attempt	1st	28	44.44
	2nd	27	42.86
	3rd	6	9.52
	4th+	2	3.17
Parent Qualification	Illiterate	3	4.76
	Literate	17	26.98
	Graduate	24	38.10
	PG	17	26.98
	PhD	2	3.17
Parent Profession	Doctor	5	7.94
	Teacher	4	6.35
	Job	16	25.40
	Business	16	25.40
	Retired	9	14.29
	Agriculture	5	7.94
	Other	8	12.70
Family Income (INR)	<10L	37	58.73
	10–20L	22	34.92
	20–50L	3	4.76
	>50L	1	1.59
Head of Family	Father	43	68.25
	Mother	5	7.94
	Self	9	14.29
	Brother	4	6.35
	Spouse	2	3.17
Doctor in Family	Yes	12	19.05
	No	51	80.95

The financial and social influences on the choice of super specialty. A large proportion required early financial returns post-PG 54 (85.71%), with most feeling that the two-year bond would influence their decision 46 (73.02%), and approximately half reported having loans or liabilities 32 (50.79%) (Table 2).

**Table 2:** Financial & Social Influencers on Super Specialty Choice.

Variable	Categories	Frequency (n)	Percentage (%)
Financial Requirement Post-PG	Early	54	85.71
	Late	9	14.29
2-year Bond Affects Decision	Yes	46	73.02
	May be	15	23.81
	No	2	3.17
Loan/Liabilities Present	Yes	32	50.79
	No	31	49.21

The most frequently cited expectations were spending time with family 39 (61.90%), reduced work pressure 30 (47.62%), and maintaining social relationships 25, 39.68%). Less working hours 17 (26.98%), no night calls 11 (17.46%), and a better quality of life 6 (9.52%) were reported less frequently (Table 3).

**Table 3:** Career & Lifestyle Expectations.

Career Expectation	Frequency (n)	Percentage (%)
Less Work Pressure	30	47.62
Less Working Hours	17	26.98
Better Quality of Life	6	9.52
Suitability to Personality	1	1.59
Social Relationship	25	39.68
Time with Family	39	61.90
No Night Calls	11	17.46

The willingness to pursue a super specialty shows that most participants 47 (74.60%) expressed willingness, 6 (9.52%) said no, and 9 (14.29%) were unsure (Table 4).

**Table 4:** Willingness to Pursue Super Specialty.

Response	Frequency (n)	Percentage (%)
Yes	47	74.60
No	6	9.52
Can’t Say	9	14.29

Seniors were the most common source, 29 (46.03%), followed by self-motivation, 18 (28.57%), teachers, 8 (12.70%), colleagues, 5 (7.94%), and family, 3 (4.76%) (Table 5).

**Table 5:** Sources of Career Guidance.

Source	Frequency (n)	Percentage (%)
Seniors	29	46.03
Self	18	28.57
Teachers	8	12.70
Colleagues	5	7.94
Family	3	4.76
Total	63	100.00

Critical care was the most preferred 26 (41.27%), followed by cardiac anesthesia 7 (11.11%), pain medicine 6 (9.52%), plastic surgery 3 (4.76%), neurosurgery 3 (4.76%), and several others, including gastroenterology, cardiovascular and thoracic surgery (CVTS), rheumatology, nephrology, cardiology, and urology (each ≤3.17%) (Table 6).

**Table 6:** Preferred Super Specialty Courses (Among Those Who Opted).

Super Speciality	Frequency (n)	Percentage (%)
Critical Care	26	41.27
Cardiac Anaesthesia	7	11.11
Pain Medicine	6	9.52
Plastic Surgery	3	4.76
Neuro Surgery	3	4.76
Neuro Anaesthesia	2	3.17
Oncology	2	3.17
Paediatric Anaesthesia	2	3.17
Gastronterology	2	3.17
CVTS	2	3.17
Rheumatology	2	3.17
Nephrology	1	1.59
Endocrinology	1	1.59
Cardiology	1	1.59
Neurology	1	1.59
Onco Surgery	1	1.59
Urology	1	1.59

The following table presents the reasons for opting for super-specialty care. More recognition 20 (31.75%) was the leading reason, followed by interest in academics 10 (15.87%), more earnings 7 (11.11%), patients’ preference for super specialists 5 (7.94%), and the challenging nature of the field 5 (7.94%). Fewer cited insufficient PG training 4 (6.35%), separation of the SS part in PG 2 (3.17%), or lack of experts 1 (1.59%) (Table 7).

**Table 7:** Reasons for Opting for Super Speciality (Among 'Yes').

Reason	Frequency (n)	Percentage (%)
More Recognition	20	31.75
More Earning	7	11.11
Interest in Academics	10	15.87
Patients prefer to go to a super specialist	5	7.94
Post-graduation training was not sufficient	4	6.35
Reframe the very challenging nature of this field	5	7.94
The super specialty part is not dealt with in PG due to separation	2	3.17
Lack of experts in the field	1	1.59

In response to the question "whether participants had a sibling/relative with super specialization," only 13 (20.63%) answered “Yes,” while 50 (79.36%) said “No.” (Table 8).

**Table 8:** Presence of Sibling or Relative with Super Specialization.

Response	n = 63	%
Yes	13	20.63
No	50	79.36

The reasons for not opting for super specialty among the “No” group, long duration was most cited 6 (9.52%), followed by financial burden 4 (6.34%), family responsibilities 3 (4.76%), late earning 2 (3.17%), and lack of interest in further study 1 (1.58%) (Table 9).

**Table 9:** Reasons for Not Opting for Super Speciality (Among 'No').

Reason	Frequency (n = 17)	Percentage (%)
Long Duration	6	9.52
Financial Burden	4	6.34
Family Responsibilities	3	4.76
Late Earning	2	3.17
Not Interested in Further Study	1	1.58
Study Place Uncertainty	1	1.58
Unmarried	0	0

The following were the perceived benefits of super specialization on a "Likert scale" [3]. The most agreed-upon benefits were gaining deeper knowledge/expertise, 25 (39.68%) strongly agree, 16 (25.39%) agree, and enhancing problem-solving skills, 25 (38.09%) strongly agree, 16 (25.39%) agree. Many agreed that it increases career earning potential 17 (26.98%) strongly agree, 20 (31.74%) agree, and professional satisfaction 17 (26.98%) strongly agree, 18 (28.57%) agree, although some responses were neutral or disagreed (Table 10).

**Table 10:** Perceived Benefits of Super Specialisation (Likert Scale 1–5).

	Increases career earning potential	Improves job market competitiveness	Gain deeper knowledge/ expertise	Enhance problem-solving skills	Increases professional satisfaction
Strongly Disagree	4 (6.34)	4 (6.34)	3 (4.76)	4 (6.34)	5 (7.93)
Disagree	6 (9.52)	5 (7.93)	4 (6.34)	5 (7.93)	10 (15.87)
Neutral / Neither Agree nor Disagree	16 (25.39)	18 (28.57)	15 (23.80)	14 (22.22)	13 (20.63)
Agree	20 (31.74)	15 (23.80)	16 (25.39)	16 (25.39)	18 (28.57)
Strongly Agree	17 (26.98)	21 (33.33)	25 (39.68)	24 (38.09)	17 (26.98)

The following were found to be the perceived drawbacks of super-specialization. A significant proportion agreed it is time-consuming/expensive, 12 (19.04%) strongly agree, 21 (33.33%) agree, and may limit job opportunities, 8 (12.69%) strongly agree, 18 (28.57%) agree. Other concerns included hindering adaptability, a narrower focus, and no guaranteed satisfaction, with many participants expressing neutrality or disagreement (Table 11).

**Table 11:** Perceived Drawbacks of Super Specialisation, Likert Scale (1–5).

	Limit job opportunities	Hinder adaptability	Time-consuming/ expensive	Narrower focus/less knowledge	No guaranteed satisfaction
Strongly Disagree	5 (7.93)	8 (12.69)	7 (11.11)	10 (15.87)	6 (9.52)
Disagree	13 (20.63)	21 (33.33)	6 (9.52)	13 (20.63)	17 (26.98)
Neutral / Neither Agree nor Disagree	19 (30.01)	20 (31.74)	17 (26.98)	22 (34.92)	20 (31.74)
Agree	18 (28.57)	8 (12.69)	21 (33.33)	12 (19.04)	11 (17.46)
Strongly Agree	8 (12.69)	6 (9.52)	12 (19.04)	6 (9.52)	9 (14.28)

The association between demographic factors and the choice of super specialty is shown below. No statistically significant associations were found between the choice of SS and gender (p = 1.00), income (p = 0.386), presence of doctors in the family (p = 0.739), or branch of study (p = 0.590) (Table 12).

**Table 12:** Association of Demographic Factors with SS Choice (Chi-Square Analysis).

Variable	Categories	% Chose SS	p-value
Gender	Male / Female	75.0 / 74.2	1.000
Income	Low / Mid / High	78.4 / 63.6 / 100.0	0.386
Doctors in Family	Yes / No	66.7 / 76.5	0.739
Branch	Medicine / Anaesthesia / Surgery	68.3 / 84.2 / 100.0	0.590

The thematic analysis of open-ended feedback from participants regarding super specialization shows three main themes. Financial barriers - Some respondents felt that the current system favours those with higher financial means, as reflected in the quote: “The System is made so that only the rich can pursue SS”, academic interest - A recurring suggestion was to increase academic exposure during PG to foster interest in super specialization: “More academics should be included in PG to motivate SS”, and systemic limitation - Policy-related and workload challenges were highlighted as deterrents: “Bond policy and work overload deter SS pursuit” (Table 13).

**Table 13:** Thematic Analysis of Open-ended Feedback.

Theme	Illustrative Quote
Financial Barriers	“The system is made so that only the rich can pursue SS.”
Academic Interest	“More academics should be included in PG to motivate SS.”
Systemic Limitation	“Bond policy and work overload deter SS pursuit.”

## Discussion

Studies on career decision-making among residents highlight the role of personal values. This study examined the perceptions of postgraduate (PG) students in medicine, surgery, and anaesthesiology regarding the pursuit of super-specialty (SS) training. The results reveal a complex interaction of demographic, financial, lifestyle, and institutional factors influencing career choices, broadly consistent with prior research in India and abroad. The fundamental skills required for clinical practice appear to be well understood by advanced students. To help students make a realistic choice of a specialty for postgraduate training, medical schools should assist their students in matching their impressions of the competencies required for particular specialties with the specialty-specific needs [4].

Demographics and background factors revealed a balanced gender distribution and a predominantly first-generation composition of medical professionals. Academic performance was modest for many, with nearly half clearing the NEET on their first attempt; yet, SS aspirations remained high. This suggests that academic excellence alone does not dictate specialization decisions, echoing Dattner et al., who noted the importance of personal values and specialty characteristics over performance metrics [1].

Financial pressures were among the strongest barriers to progress. Most participants emphasized the need for early financial returns, while half reported loans or liabilities. Bond obligations were particularly unpopular, with two-thirds viewing them as deterrents. These concerns align with those of Bhattacharya et al., who reported persistent SS seat vacancies due to financial viability, litigation risks, and a lack of institutional incentives [5].

Lifestyle preferences also strongly shaped choices. Participants prioritized family time, reduced workload, and emotional well-being over economic drivers. This explains the appeal of critical care, cardiac anaesthesia, and pain medicine among anaesthesiology postgraduate students, who are perceived to offer structured working hours. Similar patterns were noted by Das et al., who found that lifestyle preferences and self-esteem were primary motivators for pediatric PGs [2].

Despite systemic barriers, overall willingness to pursue SS was high (74.6%), though married participants-particularly women-were less inclined, reflecting family responsibilities. Importantly, no significant association was observed between SS aspirations and factors such as income, PG branch, or medical family background, highlighting the influence of intrinsic motivation and systemic factors.

Motivators included recognition and academic interest, with financial gain ranking lower in importance. Barriers such as prolonged training, financial strain, and family obligations mirrored findings from Mohan et al., who emphasized systemic gaps in postgraduate support [6]. Participants acknowledged benefits such as enhanced expertise, problem-solving ability, and satisfaction, but concerns over time, expense, and limited opportunities created ambivalence. This resonates with Shetty et al., who underscored the role of mentorship and institutional guidance in career decision-making [7]. The attitudes of employed nurse practitioners (NPs) and physician assistants/associates (PAs) are generally positive toward the concept of postgraduate specialty-specific training [8].

There was a change in students' perceptions at the end of graduation as compared to the time of entry. Their focus shifted from being in a respectful profession and serving the community to getting jobs and earning money [9]. Specialty preferences were aligned with PG background: anaesthesiology PGs chose critical care and related subspecialties, surgery PGs leaned toward plastic surgery and neurosurgery, while medicine PGs expressed diverse interests. The majority of the students preferred only the clinical subjects. Preference for branches among graduates is influenced by age-group, sex, and the type of medical school they graduate from [10]. These patterns parallel national trends favoring lifestyle-friendly or “end-branch” specialties over infrastructure-heavy fields. Laishram et al. concluded that the two main factors influencing a medical specialty's decision were interest in the field and compassion for patients [11]. Clinical branches remain preferred over non-clinical specialties, and research remains a low priority among students. Introducing innovative methods in teaching pre and paraclinical subjects and making medical teaching a more attractive career prospect can help improve this picture [12]. Despite their discontent with the infrastructure, the majority of alumni found their residency training to be advantageous for their professions [13]. Various factors like gender, marital status, friends, and seniors' advice affect the choices for preferred specialty. Various factors like gender, marital status, and friends' and seniors' advice affect the choices for preferred specialty [14]. The various groups have different factors that impact the selection of surgical subspecialties. When creating programs and organizing professional career guidance and counseling, this information can be helpful [15]. The decision to pursue a super specialty course is a complex one for medical postgraduates, and their perceptions play a significant role in the process. Several factors can influence their perception, including career aspirations, job opportunities, work-life balance, and financial considerations.

Limitations

This study has certain limitations. A small sample size is one of the limitations. Further study with a larger sample size will give more detailed information. Being a single-institution study, the findings may have limited generalizability to other settings. The cross-sectional design restricts the ability to capture evolving perceptions over time. Additionally, since the data were self-reported, the possibility of response bias cannot be ruled out. Also, the questionnaire was peer validated, which further has its own limitations.

## Conclusions

In conclusion, despite Indian PGs' strong desire for SS training, their pursuit is hampered by a lack of supervision, lifestyle concerns, and budgetary constraints. Reducing financial obstacles, filling mentorship shortages, and providing flexible pathways could increase adoption and better match the SS workforce with India's changing healthcare needs.

The recommendations that follow are based on the observations mentioned above. Establish structured career counseling and mentorship during PG training, provide financial assistance schemes and flexible bond policies, and enhance PG-level academic exposure and research opportunities. National-level policies should address systemic inequities to optimize the uptake of SS.

## Figures and Tables

**Figure 1:**
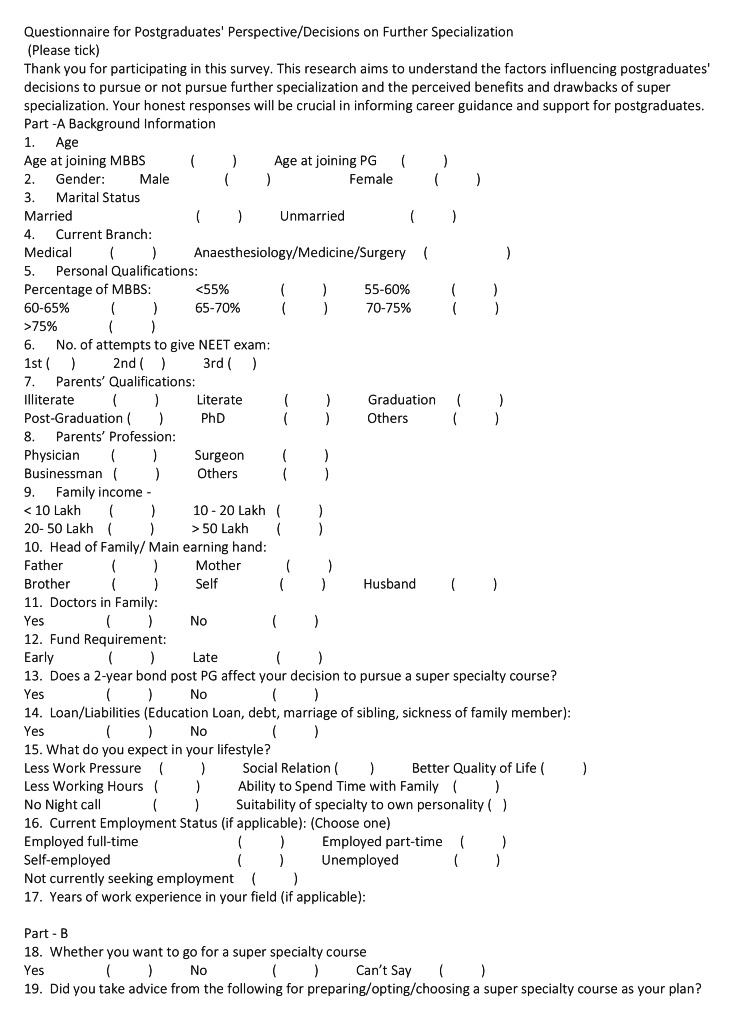
Questionnaire for Postgraduates' Perspective/Decisions on Further Specialization - Part One.

**Figure 2:**
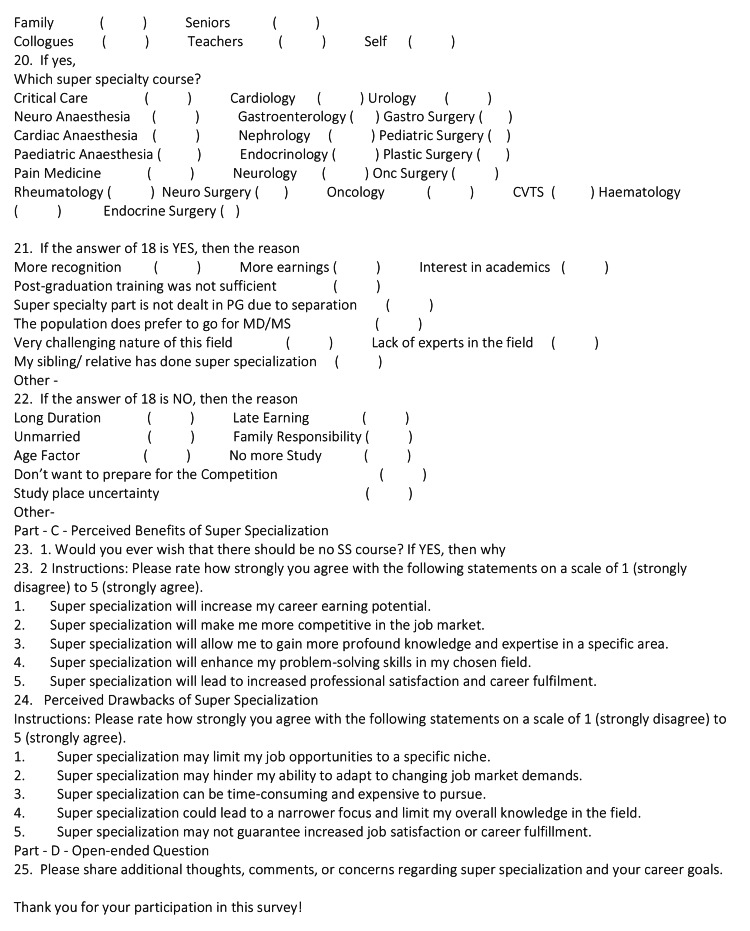
Questionnaire for Postgraduates' Perspective/Decisions on Further Specialization - Part Two.

## Appendices

Appendix 1

Appendix 2 
